# Pre-saccadic perception: Separate time courses for enhancement and spatial pooling at the saccade target

**DOI:** 10.1371/journal.pone.0178902

**Published:** 2017-06-14

**Authors:** Antimo Buonocore, Alessio Fracasso, David Melcher

**Affiliations:** 1 Werner Reichardt Centre for Integrative Neuroscience, Tübingen University, Tübingen, Germany; 2 Hertie Institute for Clinical Brain Research, Tübingen University, Tübingen, Germany; 3 Spinoza Center for Neuroimaging, Amsterdam Zuidoost, Netherlands; 4 Radiology, Imaging Division, University Medical Center Utrecht, Utrecht, Netherlands; 5 Center for Mind/Brain Sciences, University of Trento, Rovereto, Italy; University of Melbourne, AUSTRALIA

## Abstract

We interact with complex scenes using eye movements to select targets of interest. Studies have shown that the future target of a saccadic eye movement is processed differently by the visual system. A number of effects have been reported, including a benefit for perceptual performance at the target (“enhancement”), reduced influences of backward masking (“un-masking”), reduced crowding (“un-crowding”) and spatial compression towards the saccade target. We investigated the time course of these effects by measuring orientation discrimination for targets that were spatially crowded or temporally masked. In four experiments, we varied the target-flanker distance, the presence of forward/backward masks, the orientation of the flankers and whether participants made a saccade. Masking and randomizing flanker orientation reduced performance in both fixation and saccade trials. We found a small improvement in performance on saccade trials, compared to fixation trials, with a time course that was consistent with a general enhancement at the saccade target. In addition, a decrement in performance (reporting the average flanker orientation, rather than the target) was found in the time bins nearest saccade onset when random oriented flankers were used, consistent with spatial pooling around the saccade target. We did not find strong evidence for un-crowding. Overall, our pattern of results was consistent with both an early, general enhancement at the saccade target and a later, peri-saccadic compression/pooling towards the saccade target.

## Introduction

In complex scenes, high-acuity visual processing is limited to the fovea while information in the periphery is processed in less detail. We are typically not aware of these fundamental limits because we overtly shift our focus of attention and visual processing several times per second using saccadic eye movements. These overt shifts of attention, that underlie a change in fixation position, have been reported to be more efficient than covert shifts of attention (i.e. without eye movement execution) in a variety of psychophysical tasks. One clear example of an improvement in performance for saccades, compared to a covert attention shift, is when crowded displays are used as stimuli (i.e. visual crowding, [1], [2]). Directly fixating the target, via a saccadic eye movement, is more effective than trying to resolve it in peripheral vision using covert attention. Crowding by nearby items leads to the impression of being aware of a target but not being able to resolve and separate its features from nearby flanking items [1]. There are several theories about the mechanisms and sites of crowding, with converging evidence that it may take place at multiple stages of visual processing [2], [3].

A number of mechanisms have been proposed in which the preparation of a saccadic eye movement may change performance in discriminating a peripheral target even before the start of the movement itself. Perhaps the most powerful effect is the transient reduction in visual sensitivity during the eye movement itself, called saccadic suppression [4], [5]. In addition, however, during the pre-saccadic time interval the deployment of selective spatial attention is tightly linked to the saccade target and this can lead to enhanced performance at that location at the expense of nearby locations [6], [7], [8]. Unlike a covert shift, in which the oculomotor system and the attention system might have differing priorities in what is effectively a divided attention task, an overt shift of attention optimizes processing resources to a single location congruent with the saccade goal. This stronger coupling between the overt and covert shift has been shown to influence behavior in different ways. A classic example is when a target surrounded by distractors is displayed either at the saccade goal or in another location away from the saccade goal [6], [8]. When the location of the discrimination target and the saccadic target were coincident, the performance was improved compared to a condition in which the two locations were dissociated in space. Similarly, Deubel [9] proposed that the time course of this attentional enhancement is mainly flat during the pre-saccadic period when target location is predictable. Moreover, performance has been shown to not only be enhanced at the target location during saccade preparation but it also suppressed at nearby locations [8]. More recently, it has been argued that saccade preparation not only enhances the saccade target [10], [11], but even influences the spatial frequency and orientation tuning of visual processing at that location [12].

Second, from a neurophysiological perspective, during the peri-saccadic time period many neurons change their spatial and temporal selectivity based on the impending saccade in a process called “remapping” [13]. The remapping of receptive fields shows a complex pattern of reorganization, whereby the neurons can change their spatial tuning in line with the saccade direction, i.e. “predictive remapping” [13], [14], [15], or towards the saccade target, i.e. spatial compression of receptive fields [16], [17]. Many neurons also show temporal shifts in neural responses [14], [15], [18]. In the case of compression, many neurons appear to respond to stimuli at nearby locations as if they were presented at the fovea. This change in spatial selectivity would suggest that the neural response to stimuli in the peri-saccadic time period would actually be a combination of the responses to multiple items near the saccade target.

Recently, it has been suggested that these two mechanisms, allocation of increased processing resources (selective attention) and remapping of receptive fields, are present in individual neurons in the primate cortical area V4 but showing different time courses and supporting different functions [19], [20]. This finding is of considerable theoretical and practical interest, since previous behavioral and neurophysiological studies have used a wide variety of different experimental paradigms in terms of number, type, location and duration of the stimuli used and have yielded wildly different conclusions (for review: see [21], [22]). The existing literature does not fit well into a single mechanism or a single explanation for visual stability. The finding of two different mechanisms, with different time courses, might provide a future organizing principle to help link different neural mechanisms to specific aspects of trans-saccadic perception. For example, the predictive remapping of receptive fields towards their future locations could help to maintain perceptual stability of non-target objects [23], while compression of receptive fields toward the saccadic target might instead be associated with attentional allocation and enhancement of the saccadic target [17].

In laboratory studies, however, it is often difficult to disentangle effects associated with attentional allocation and spatial compression, since the focus of selective attention and the center of spatial compression coincide at the saccade target prior to saccades [17], [24]. In other words, participants might do better/worse in a particular task because more/fewer resources are allocated at that location, or instead because of the peri-saccadic spatial compression observed during the peri-saccadic interval.

In the current study, we used a combination of spatial crowding and temporal masking to try to distinguish between the time courses and effects of these two types of peri-saccadic changes in visual processing. Crowding is perhaps the best example of the difference between covert and overt attention shifts, since saccades are much more effective in discriminating the target than a covert attention shift since the saccade allows the target to be viewed with the high-resolution fovea (but see: [25], [26]). Recent studies have reported that making an eye movement influences crowding even before saccade onset [27], [28], [29], [30]. Although those studies put forward the hypothesis that signals associated with saccade preparation might change the spatial resolution for a target presented around saccadic location, the design of those studies, which measured improvement in performance at the saccade target, could not distinguish between a more general benefit of pre-saccadic focusing of processing resources (enhancement) and effects driven specifically by the spatial compression of receptive fields during the pre-saccadic interval [17]. Moreover, it has been reported that even covert attention at the target location, without the need to move the eyes could enhance discrimination performance in crowding environment [25], consistent with a possible role of enhancement rather than remapping.

In the present set of experiments, we used two main manipulations to characterize effects that might be related to peri-saccadic compression and attentional effects of saccades on processing at the saccade target. First, we included trials with and without masking. We followed this procedure since previous studies interested in the effect of eye movement preparation upon crowding made use of such masked stimuli [27] and we aimed to replicate the baseline effects with a similar display. One hypothesis could be in fact that the combination of crowding and masking in previous studies may have created a form of “super-masking” [31] and so the effects of saccades on temporal perception [32], [33] and masking [34], [35] could have masqueraded as spatial effects (see: [36]). Pre-saccadic perception can in fact “misplace” the location of a probe (relative to the mask) toward the saccadic target, unmasking the probe stimulus and consequently increasing discrimination performance [34], [35]. Indeed, a recent paper using crowded letter stimuli found that any effect of saccades on performance was better explained by “unmasking” rather than “uncrowding” [37]. For example, those authors reported that pre-saccadic enhancement in performance was found also for un-flanked letter targets followed by a mask, consistent with a temporal rather than a spatial effect. Moreover, in a similar experiment to Harrison et al. [27], Ağaoğlu and Chung [38] reported that during the pre-saccadic period the level of crowding was not in fact reduced compared to a fixation condition when different combination of high/low contrast target/flanker stimuli were used. On the contrary, saccades actually made crowding worse in some of the conditions tested. These results are not consistent with un-crowding, but suggest instead that peri-saccadic effects are linked to previously reported mechanisms such as saccadic suppression and peri-saccadic mis-localization.

Second, we varied the orientation of the flankers across trials in order to measure spatial pooling (a possible consequence of peri-saccadic spatial compression) and its time course. One of the main explanations for crowding is that features from the flankers are pooled with those of the target. According to the pooling account, even when observers cannot report the target orientation they are still able to report the mean of the configuration, suggesting that crowding induces compulsory pooling of the flanker elements and the target [39]. This pooling effect would be expected to be strong in the case when the average orientation of the flankers is quite different than the orientation of the target. In contrast, when the flankers are all vertical, studies have shown that this can induce configural effects, potentially making the task easier. It is well known that when the flankers share some characteristics, like orientation, they can be grouped, reducing the crowding effect [40], [41], [42], [43], [44], [45], [46]. For these reasons, we directly compared the discrimination performance during fixation trials when the flankers were all oriented vertically (as in [27]) against a condition in which the flankers had randomized orientation for both no-mask and mask trials, reducing possible higher level configural/pop-out effects of the target with respect to the flankers.

Manipulating the orientation of the flankers also allowed us to directly compare the predictions of two different potential mechanisms that might be involved in peri-saccadic effects on crowding: spatial compression and receptive field shrinking. Spatial compression of receptive fields towards the saccade target [17], [24] should lead to greater feature pooling since features from the distractors would be shifted towards the target, creating spatial imprecision of responses in neurons with nearby receptive fields (see, for example, [24] Fig 2c). One potential outcome of this loss of spatial selectivity is that multiple items would, effectively, fall into the same receptive fields. Behavioral studies have also repeatedly found that stimuli flashed around the time of saccades are perceived as compressed towards the saccade target (for review, see: [32]). This makes the specific prediction that, shortly before the saccade, participant responses should follow the average orientation of all the flankers near the saccade target. When the average orientation was the same as the target, then participants would respond correctly, but when the average flanker orientation was opposite of the target then they should actually perform worse than chance level.

In contrast, it has been argued that immediately before the saccade, receptive fields around the saccadic target might effectively shrink, either due to becoming more spatially selective at the saccade target [16] or, instead, “remapping” against the direction of the saccade to respond as if they were foveal receptive fields [28]. If so, then performance should improve regardless of flanker orientation since spatial precision at the saccadic target should increase and thus pooling would be reduced.

## Methods

### Participants

A total of 32 volunteers (26 females; Experiment 1: six; Experiment 2A: six; Experiment 2B: eight; Experiment 3: six; Experiment 4: six) aged between 18 and 30 years participated in the study. All reported to be free from neurological and visual impairments. Written informed consent was obtained from all subjects, in accordance with the 1964 Declaration of Helsinki. The protocol was approved by the University of Trento Research Ethics Committee. All participants received a small compensation of €7 per testing hour.

### Apparatus

Stimuli were presented on a 19-inch CRT monitor (1024 x 768 pixels) at 100 Hz. Participants were seated with their head resting on a chin and forehead rest in order to reduce head movements. The eyes were horizontally and vertically aligned with the center of the screen at a distance of 62 cm. Eye movements were recorded with the EyeLink 1000 system (detection algorithm: pupil and corneal reflex; 1000 Hz sampling; saccade detection was based on a 30 deg/s velocity and 9500 deg/s^2^ acceleration thresholds). Participant responses in the orientation perception task were recorded on a standard keyboard. A five point-calibration on the horizontal and vertical axes was run at the beginning of each experimental block. The programs for stimulus presentation and data collection were written in MATLAB (MathWorks) using the Psychophysics Toolbox Version 3 [47], [48], and Eyelink Toolbox extensions [49].

### General procedure and stimuli

**Fig 1:**
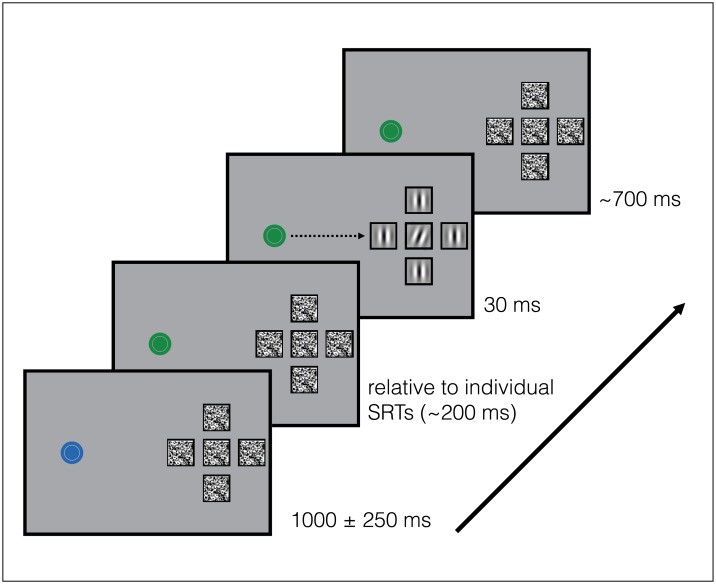
Experimental design and stimuli configuration. Panel A. Trial sequence of Experiments 1–3. Participants were instructed to maintain fixation and to make a saccade towards a square appearing to the right side of the fixation cross at 7.7° of eccentricity as soon as the central dot turned green. Participants reported the orientation of a Gabor patch presented for 30 ms and tilted either clockwise or counterclockwise. In mask trials, patches of white noise were displayed at each refresh rate filling in the placeholders before and after the Gabor presentation. In the current figure, a mask trial is depicted with the flankers all set as vertical, as in Experiments 1 and 2 (2 also included a random orientation condition). In Experiment 3 and 4, the flankers were constructed in a way that if the central Gabor was oriented to the left, the average of the orientation of the flankers was oriented to the right (and vice-versa). We manipulated the mean orientation so that it spanned from -20 to +20 degrees. Stimuli are not drawn to scale, but rather to illustrate the configuration.

The general design of the study followed the methods and procedure of Harrison and colleagues [27], with the addition of a few changes in order to distinguish between the attentional, spatial and temporal aspects of the effect of saccades on perception. In all of the experiments, stimuli were presented on a uniform grey background. Participants started each trial by pressing the space bar while maintaining their gaze at the central fixation dot (blue circle, 0.25 degrees of visual angle—dva). At the start of the trial, five square placeholders (side length 1dva) appeared in the periphery at approximately 7.7 dva of eccentricity, with respect to the central fixation dot, on the right side of the screen (Fig 1). On each trial, the configuration was jittered by ± 0.4 dva to limit eye movement pre-programming. The distance between the placeholders varied according to the experimental condition in order to change the flanker distance in the crowding display (see details below). After a random interval varying between 750 and 1250 ms, the fixation dot turned green signaling either to covertly attend the central placeholder (fixation condition blocks) or to make an eye movement toward the central placeholder (saccade condition blocks). After a variable interval (computed as described below), five Gabor patches (Gaussian windowed spatial frequency gratings; width: 1dva, 2 cpd, 100% contrast) were presented for 30 ms inside the placeholders. At the end of each trial, participants were required to report the orientation of the central Gabor stimulus, randomly tilted 15° or 18° to either the left (counter-clockwise from vertical) or to the right (clockwise from vertical). Participant were instructed to press the key labeled “1” on the keypad for left and the key labeled “2” for right.

The other four patches, *flankers*, were presented either with a vertical orientation (Experiments 1 and 2; see: [27]) or with differing orientations (Experiments 2, 3 and 4). In Experiments 1, 2 and 3, *no-mask* trials were intermingled with *mask* trials in equal proportion, while in Experiment 4 only no-mask trials were presented. During no-mask trials the placeholders remained empty throughout the trial except during target and flanker presentation, when the Gabor patches were briefly presented. In mask trials, patches of white noise were displayed at each refresh rate (every 10ms) filling in the placeholders before and after the Gabor presentation (as in [27]). On separate blocks of trials, participants were required either to maintain fixation (Experiments 1A, 3A and 4) or to make a saccade toward the configuration (Experiments 1B, 3B and 4). The order of running in the two sub-experiments (fixation or saccade) was counterbalanced across participants in order to avoid any order effects.

In saccade blocks, in order to optimize the timing of the presentation of the Gabor during the peri-saccadic interval, the onset of the stimulus from green fixation dot was calculated by randomly subtracting 150, 100 or 50 ms from the estimated median saccadic reaction time. We ran a drift correction after every 20 trials in order to check that the gaze was aligned to the center of the screen. In fixation blocks, the onset of the stimulus was 100, 150 or 200 ms after green fixation dot. A detail description of each experiment and its conditions is provided in separate subsections below.

### Statistical analysis

Data were analyzed using the R software for statistical computing [50]. For each experiment, all the data were pooled together across participants and analyzed by means of bootstrapping [51] using participants as different strata. We created each bootstrap sub-dataset by sampling with replacement 2000 times. In all the experiments, we reported the 95% of bootstrap confidence interval (CI) of the parameters describing the best fit of the data along the dimension of interest. The data were analyzed with a General Linear Model, with the formulation proposed by McCullagh and Nelder [52], using the logit function as link function (logistic regression model):

$$logit\left(p\right)=log\left(\frac{p}{\left(1-p\right)}\right)$$ (1)

The estimated mean and confidence intervals were derived for each experimental condition by calculating the inverse of the logit function (logistic function).

$$logistic\left(y\right)=\frac{1}{\left(1+e^{-y}\right)}$$ (2)

### Eye movement analysis

In Experiments 1B, 3B and 4, we first calculated the timing of the Gabor presentation with respect to saccadic onset. To assure that the Gabor was presented while the eyes were at rest and to make it possible to directly compare with the fixation condition, we subtracted the start of the saccade from the Gabor offset. According to this convention, negative values represented stimuli that were presented before the onset of the eye movement. Only the data in which the saccade started after Gabor offset were included in the analysis. In a second step, we divided the data into four temporal bins during the pre-saccadic interval, plus the fixation condition. We ran two bootstrapping models, with one predictor for each of the mask conditions. In the first, we estimated the overall contribution of the eye movement compared to the fixation condition. In the second, we estimated the performance across the pre-saccadic interval of different time bins. For all of the analyses, the alpha level was corrected for each test according to the number of comparisons (Bonferroni corrected). The 95% CI of each bootstrapped GLM fit for each experiment and condition of interest are reported in Table 1. Saccade trials in which there were saccades with a latency of less than 80 ms or more than 500 ms and with amplitudes smaller than 4.5 dva were excluded.

**Table 1:** Estimated upper and lower confidence interval for each experiment and condition.

Experiment	Fit condition			
1A		***Mask***	***TFD***	Mask*TDF
		[-0.99 -0.31]*	[0.25 0.49]*	[-0.36 -0.10]*
1B		***Saccade vs Fixation***		
		***TFD = 1*.*5dva***	***TFD = 2*.*0dva***	***TFD = 3*.*5dva***
	no-mask	[0.33 1.02]*	[0.50 1.29]*	[-0.21 0.75]
	mask	[-0.08 0.45]	[0.16 0.68]*	[0.11 0.69]*
			***pre-saccadic***	
	no-mask	[-0.45 0.08]	[-0.14 0.41]	[-0.31 0.28]
	mask	[-0.08 0.20]	[-0.03 0.24]	[-0.21 0.10]
2A		***TFD***		
		***Flankers vertical***		***Flankers random***
	no-mask	[0.20 0.64]*		[0.11 0.45]*
	mask	[-0.08 0.24]		[-0.39 0.36]
2B	no-mask			[0.13 0.25]*
	mask			[0.15 0.29]*
3A		**Flanker angle**		
		***TFD 1*.*2dva***	***TFD 2dva***	***TFD 5dva***
	no-mask	[-0.48 -0.20]*	[-0.32 -0.07]*	[-0.06 0.25]
	mask	[-0.65 -0.36]*	[-0.28 -0.02]*	[-0.36 -0.07]*
3B		***Saccade vs Fixation***		
		***flanker angle 6°***		***flanker angle 18°***
	no-mask	[0.05 0.56]*		[0.15 0.64]*
	mask	[-0.05 0.51]		[-0.07 0.42]
		***pre-saccadic***		
	no-mask	[-0.34 -0.04]*		[-0.13 0.12]
	Mask	[-0.03 0.23]		[-0.10 0.13]
4		***Saccade vs Fixation***		
		***flanker angle 6°***		***flanker angle 18°***
	congruent	[-0.37 0.10]		[-0.60 -0.12]*
	incongruent	[-0.56 -0.07]*		[-0.89 -0.37]*
		***pre-saccadic***		
	congruent	[-0.28 -0.02]*		[-0.28 -0.04]*
	incongruent	[-0.17 0.15]		[-0.20 0.05]
4		***Saccade vs Fixation***		
		***flanker angle 6°***		***flanker angle 18°***
	congruent	[-0.37 0.10]		[-0.60 -0.12]*
	incongruent	[-0.56 -0.07]*		[-0.89 -0.37]*
		***pre-saccadic***		
	congruent	[-0.28 -0.02]*		[-0.28 -0.04]*
	incongruent	[-0.17 0.15]		[-0.20 0.05]

## Experiment 1

In the first experiment, we measured orientation discrimination for crowded targets with and without saccades and with and without a backward mask. The main purpose was to try to replicate the initial report that saccades influenced performance in this task [27].

## Experiment 1: Methods

### Experiment 1A—Fixation

In Experiment 1A, which measured the influence of masking and flankers on orientation discrimination, participants were required to refrain from making eye movements toward the target Gabor. We varied the presence of the *mask* (no-mask vs. mask) and we manipulated the distance of the flankers relative to the central target (TFD: target-flanker-distance). In particular, we used five different TFDs, randomly chosen on each trial between 1.2dva, 1.5dva, 2dva, 3.5dva and 5dva. In this experiment, the flankers always had a vertical orientation. Participants performed 8 blocks of trials. Each block consisted of 80 trials (40 mask and 40 no-mask trials for each target flanker distance tested), for a total of 640 trials for each participant. For the statistical analysis, the data were modeled with two predictors, *mask* (no-mask vs. mask) and target-flanker distance, plus their interaction.

### Experiment 1B—Eye movement

In Experiment 1B, all of the stimuli were identical to Experiment 1A but participants were required to make an eye movement toward the central target as soon as the fixation dot turned green. As in Experiment 1A, we varied the presence of the mask (no-mask vs. mask) and we used only three different TFDs, randomly chosen on each trial between 1.2dva, 2dva, 3.5dva of visual angle. Participants performed between 9 and 11 blocks of trials. Each block consisted of 72 trials (36 mask, 36 no-mask, 12 trials for each eccentricity tested), for a total of 648 to 792 trials for each participant. A total of 10% of trials were excluded because they did not meet our inclusion criteria (see Eye movement analysis subsection).

## Experiment 1: Results

### Experiment 1A

**Fig 2:**
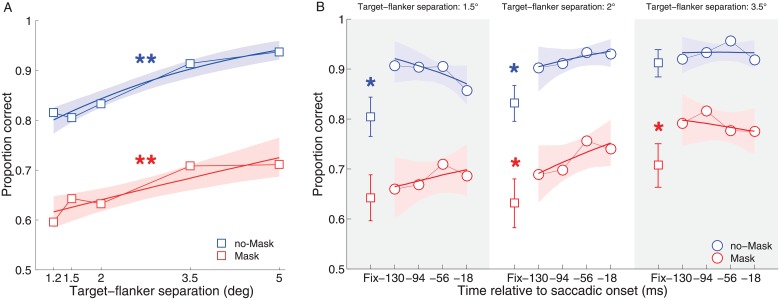
Mean performance in Experiment 1A and 1B. Panel A. Fixation condition (Experiment 1A). The squares represent the estimated mean proportion of correct responses at each target-flanker distance (TFD). Panel B. Saccade condition (Experiment 1B). The circles represent the estimated mean proportion of correct responses along the pre-saccadic interval. The first bin (square symbol) represents the mean performance at fixation and its 95% confidence interval (CI). The data are subdivided in three columns according to each TDF: 1.5 degrees of TFD (gray), 2 degrees of TFD (white), 3.5 degrees of TFD (gray). Data are displayed relative to saccade onset. In all the graphs, shaded areas represent the bootstrapped 95% CI. Continuous lines represent the GLM fit across the dimension of interest (TFD: Experiment 1A; pre-saccadic interval: Experiment 1B). Blue symbols indicate no-mask trials, while red symbols show mask trials. Single asterisks indicate a significant difference between the fixation and the saccade condition. Double asterisks indicate a significant slope across the dimension of interest.

In Experiment 1A, we tested the effect of a mask when discriminating a target among flanker elements in order to establish the baseline contribution of the mask. To do so, we varied the presence of the mask and we manipulated the distance of the flankers relative to the central target. As shown in Fig 2A, performance improved for larger TFDs, as expected, with a significant slope across target-flanker separations (95% CI: [0.25 0.49]). This result suggests that, for both the no-mask and mask condition, increasing the target-flanker distance is reflected in better discrimination performance, replicating the well-known crowding effect [1] within our setup.

As expected, we also found a strong influence of the mask upon discrimination performance (95% CI: [-0.99–0.31]) (see, for example: [31]). In the no-mask condition, performance varied between 80% to 95% correct. In contrast, adding the mask reduced performance across distances of about 20%, spanning from around 60% to 75% correct performance. Importantly, performance in the mask condition was quite similar to that reported in the previous study which we aimed to replicate [27].

### Experiment 1B

In Experiment 1B, we tested the effect of adding an eye movement on the ability to discriminate a Gabor patch surrounded by flanker stimuli at different separations. On the one hand, a previous study reported an increase in orientation discrimination performance during the pre-saccadic period (Harrison et al., 2013a) whereas another study, using letters as stimuli, argued that the effect might have been driven mainly by unmasking alone [37], (see also: [36]). To disentangle between spatial and temporal explanations, participants were required to make an eye movement toward the central target. We also varied the presence of the mask and we used three different TFDs.

Our time-course analysis allowed us to look at both the general effect of preparing a saccade, defined as the benefit between the mean performance during the pre-saccadic period and fixation trials, and any separate effects related to remapping which should be greatest near to saccade onset [17], [24], [32], [33]. Fig 2B shows the pattern of perceptual discrimination for no-mask (blue symbols) and mask (red symbols) stimuli at three different TFDs of 1.5dva, 2dva and 3.5dva of visual angle along the pre-saccadic interval. At each TFD the data are also compared with the fixation condition (the first bin on the left of each subpanel). As in Experiment 1A, we observed a main effect of the mask [37], with a difference of about 20% between the two conditions at each target flanker distance (95% CI: [-1.81, -1.41]). More interestingly, we also found a general benefit for eye movement trials compared to fixation trials, in particular in the 1.5dva and 2dva no-mask conditions (95% CI, [0.33 1.02] and [0.50 1.29], respectively) and the 2dva– 3.5dva mask conditions (95% CI, [0.16 0.68] and [0.11 0.69], respectively). Moreover, as predicted, performance scaled moderately but to a significant degree along target-flanker distances in Experiment 1B (Fig 2B). In particular, there was a clear benefit for the Mask condition when a saccade was prepared (1.5 dva: [0.62 0.71; 2 dva: [0.66 0.74]; 3.5 dva: [0.75 0.83]), similar to the increase recorded in the fixation condition (Experiment 1A) but with performance at each target-flanker distance relatively higher. On the other hand, for no-mask stimuli preparing a saccade pushed performance close to ceiling, making the specific perceptual benefit effect difficult to record, although performance still increased for larger target-flanker distances (1.5 dva: [0.87 0.94]; 2 dva: [0.88 0.94]; 3.5 dva: [0.90 0.95]). Nonetheless, despite seeing this saccade-related effect, we can not make a strong prediction about the strength of crowding itself, since we did not test performance for a no-crowded target.

More interestingly, there was not a consistent or significant change in performance along the time course within the pre-saccadic interval, suggesting that the effect was not tightly time-locked to the onset of the saccade. A similar lack of such time-course was already showed by Deubel [9] whereby the pre-saccadic enhancement for a discrimination stimulus (briefly displayed at saccade goal) was showed to be relatively flat in a similar time window as the one reported in the present paper. On three out of the six conditions, the slope was slightly positive and in the other half it was slightly negative (no significant main effect of time bin). Thus, the overall pattern of results was consistent by a general enhancement in performance for saccade compared to fixation trials, but not any change over the peri-saccadic time period.

## Experiment 1: Discussion

The first main finding was to confirm that masking did indeed have a strong effect on orientation discrimination performance in this task. This was important in order to confirm the idea that the main influence of saccades on performance are primarily due to un-masking rather than un-crowding [36], [37], [38]. Second, we found a general pre-saccadic improvement in performance (significant in 4 out of 6 conditions) that was present already quite early and remained constant throughout the time course of the pre-saccadic time period. Both of these findings were consistent with the suggestion that temporal factors (reduced masking) play a role in peri-saccadic perception of these stimuli [36], [37]. Moreover, both of these effects were quite similar for the three different flanker-to-target distances. Overall, this pattern of results was more consistent with a temporal than a spatial effect [36], [37].

However, neurophysiological studies have demonstrated that remapping has both a temporal and spatial component (for review, see: [32]). To look more carefully at the spatial interaction between the flanker and the target, we ran an additional set of experiments to measure how the flankers influenced perception in this task, both in general and during the peri-saccadic time period.

## Experiment 2

In this experiment, we systematically varied the orientation of the flankers in two conditions, either all vertical (as in Experiment 1) or with complete random orientations. As described in the Introduction, this manipulation of flankers allowed us to investigate two potential issues with the all-vertical flankers used previously. The vertical flankers may have induced some configural effect that made the task easier [40], [41], [42], [43], [44], [45], [46] or even interacted with the peri-saccadic effects, in such a way that for participants was easier to suppress flanker information [23] since they were identical to each other, leading to a general increase in performance.

This experiment was run in two different versions. In the first version, we tested performance with and without masking for both vertical and random orientations (2 x 2 design). In the second version, we replicated and extended the results using more levels of target-flanker distances and with all trials having randomized flanker orientations. In both versions, participants maintained fixation so that we could precisely measure the role of flankers in the absence of saccades, prior to examining the role in saccades in Experiments 3 and 4.

## Experiment 2: Methods

### Experiment 2A

The TFDs were set at three levels: 1.5dva, 2dva and 3.5dva. As in the previous experiments, we also varied the presence of the mask (no-mask vs. mask). Participants performed 8 blocks of trials. Each block consisted of 96 trials (48 mask, 48 no-mask, 16 trials for each tested eccentricity, 8 trials with vertical flanker orientation and 8 trials with random flanker orientation), for a total of 768 trials for each participant.

### Experiment 2B

In Experiment 2B, we tested fixation trials with and without masks, always using random flanker orientations at four levels of TFD: 1.2dva, 1.5dva, 2dva and 5dva. This second version of the experiment was run to replicate and to better characterize performance in no-mask and mask condition when random flankers were used. Participants performed between 10 and 11 blocks of trials. Each block consisted of 40 trials (20 mask, 20 no-mask trials for each tested eccentricity), for a total of 400–440 trials, for each participant.

### Data analysis

Fixation trials in which participants made an eye movement larger than 1dva were excluded (~5% of trials were discarded). In Experiment 2, data were separately modeled for no-mask and mask trials using target-flanker distance as a predictor.

## Experiment 2: Results

### Experiment 2A

**Fig 3:**
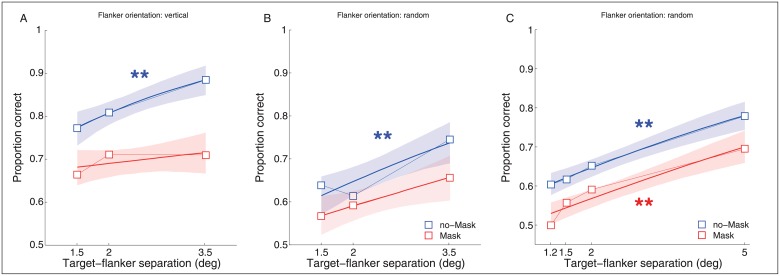
Mean performance at fixation in Experiment 2A and 2B. Squares represent the estimated mean proportion of correct responses at each TFD. Panel A. Mean performance when the flankers were all vertical (Experiment 2A). Panel B. Mean performance when the flankers were random (Experiment 2A). Panel C. Mean performance when the flankers were all random and a larger TFD range was used (Experiment 2B). All other graphical conventions (lines, colors, asterisks) are identical to those used in Fig 2.

In the vertical flanker condition, we replicated the finding of a decrease in performance for trials with the forward and backward mask (Fig 3A). The model reached significance along the TFD parameter only for the no-mask condition (95% CI: [0.20 0.64] and [0.11 0.45] for the vertical and random flanker orientation, respectively). More interestingly, when the flankers had a random orientation we found that performance dropped about 10% for both no-mask and mask conditions (95% CI, [-0.74–0.45]). This result suggests that the vertical flankers were inducing some configural effect that made the task with vertical flankers easier than with random flanker orientations [40], [41], [42], [43], [44], [45], [46].

### Experiment 2B

To confirm and better characterized the effects found in Experiment 2A, we ran a control experiment to test no-mask and mask condition across a wider range of different TFDs (1.2dva, 1.5dva, 2dva and 5dva) by always using flankers with a random orientation. As shown in Fig 3C, discrimination performance scaled with TFD for both the no-mask and mask condition (95% CI, [0.13 0.25] and [0.15 0.29], respectively). In this version of the experiment, perceptual performance ranged between ~60% and ~80% in the no-mask and it was around 10% lower for the mask condition.

## Experiment 2: Discussion

Taken together, the data of Experiments 2A and 2B showed that randomizing the flankers dramatically reduced performance, for both mask and no-mask trials. We further investigated these effects in Experiments 3 and 4 to better characterize the role of flanker orientation with and without saccades.

## Experiment 3

The results of Experiment 2 suggested that the vertical flanker orientation might have induced configural effects, in addition to just crowding. For this reason, we ran Experiment 3, in which we tested the ability to discriminate a target among flanking distracters when the mean orientation of the flankers was systematically varied. Specifically, we manipulated the average orientation of the four flankers so that the mean was always pointing to the opposite orientation compared to the central Gabor. For example, when the central Gabor was pointing to the left, the mean orientation of the four flankers would point to the right. Numerically, if the target was, for example, 18 degrees in orientation, the flankers might have been: -29, 33, -19 and -33 degrees, with a flanker average of -12 degrees in the direction opposite to the target (all angles are expressed with respect to 90 degrees). This manipulation is critical since the direction of the performance indicates if participant responses were mainly driven by the orientation of the target or, instead, by the mean orientation of the flankers (due, for example, to compulsory pooling across the target-flanker configuration). However, the individual flankers could be oriented either to the right or left, even when the mean was to the right or left, such that no individual flanker could predict the correct answer.

This experiment was divided in two conditions, run in counter-balanced order across participants. In Experiment 3A, we measured performance with maintained fixation. In Experiment 3B, we investigated the nature of spatial pooling in the pre-saccadic time period. The method was identical to the fixation condition but the same participants were now required to make an eye movement toward the target. The time course of discrimination performance was measured when the orientation of the flankers was systematically varied, with and without the mask. This allowed us to see whether the influence of the distractors varied over the time course of the pre-saccadic period.

## Experiment 3: Methods

### Procedure

In experiment 3A, we further investigated the role of flanker orientation by systematically controlling the mean orientation of the flankers. On each trial, we calculated the average orientation of the four flankers so that the mean was always pointing to the opposite orientation compared to the central Gabor. The average orientation randomly varied between ±20° of visual angle to the left or to the right. As in the other experiments, we varied the presence of the mask (no-mask vs. mask) and the TFD was randomly chosen between 1.2dva, 2dva and 5dva. Participants performed 12 blocks of trials. Each block consisted of 72 trials (36 mask, 36 no-mask, 12 trials for each target-flanker distance tested), for a total of 864 trials for each participant. Within each block, there were 12 catch trials, i.e. trials in which the flanker mean had the same orientation as the target.

Experiment 3B was identical to the fixation condition but participants were required to make an eye movement toward the target as soon as the fixation dot turned green. We restricted the experiment to just one TFD of 2 dva. The same participants tested on Experiment 3A performed 12 blocks of trials. Each block consisted of 72 trials (36 mask, 36 no-mask), for a total of 864 trials for each participant. As in Experiment 3A, within each block, there were 12 catch trials. Data were separately modeled based on mask and flanker condition and we divided the data in four mean-orientation quartiles (from 3–21 degrees of orientation).

Fixation trials in which participants made an eye movement larger than 1dva were excluded (Experiment 3A: ~5%). Saccade trials in which there were saccades with a latency of less than 80 ms or more than 500 ms and with amplitudes smaller than 4.5 dva were excluded (Experiment 3B: ~20%).

## Experiment 3: Results

### Experiment 3A

**Fig 4:**
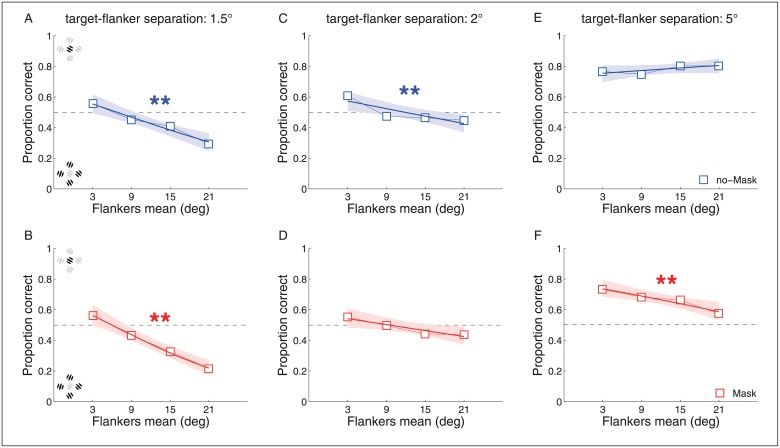
Fixation condition in Experiment 3A. The squares represent the estimated mean proportion of correct responses for each flanker mean orientation bin. Subpanels (A-B) represent 1.5 degrees of TFD, (C-D) 2 degrees of TFD and (E-F) 5 degrees of TFD. All other graphical conventions (lines, colors, asterisks) are identical to those used in Fig 2.

As expected, the mean orientation of the flankers influenced the reported orientation of the target (Fig 4). Increasing the average orientation of the flankers, which was in the opposite direction to the target orientation, decreased the ability to discriminate the target orientation. In particular, in the no-mask condition this was significant for 1.2dva and 2dva (95% CI: [-0.48–0.20] and [-0.32–0.07], respectively) while in the mask condition for 1.2dva, 2 and 5dva (95% CI: [-0.65–0.36], [-0.28–0.02] and [-0.36–0.07], respectively). This effect strongly resembled the compulsory pooling proposed by Parks and colleagues [39], as described above. In some conditions, participants were clearly responding to the average flanker orientation leading to performance well below chance. Consistent with the previous experiments, the intercept increased significantly across the three target-flanker separations for both mask and no-mask (95% CI, [0.30 0.39]) suggesting once again that separating the flankers from the target decreased the effect of crowding.

The outcome of Experiment 3A raises the possibility that if the discrimination performance is dictated by the mean orientation of the flankers (as shown in Fig 4), a similar decrement in performance might be observed during the pre-saccadic period. As described in the Introduction, it has been repeatedly observed that just prior to saccade onset, nearby items are spatially compressed towards the location of the saccadic target. Our hypothesis was that such compression of elements, immediately prior to saccades, might favor spatial pooling. Alternatively, the proposal that receptive fields might effectively shrink prior to saccade onset would predict that the effect of the flankers might be reduced along the pre-saccadic time course. If no change in performance for saccades was found compared to the fixation condition, then this would be consistent with similarly strong effects (and similar time courses) for both pooling and enhancement.

### Experiment 3B

**Fig 5:**
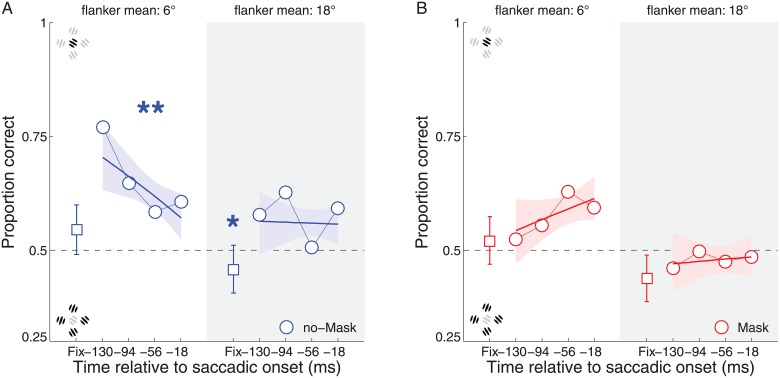
Mean performance in the saccade condition for Experiment 3B and 4. The circles represent the estimated mean proportion of correct responses along the pre-saccadic interval. The first bin (square symbol) represents the mean performance at fixation and its 95% CI. The data are subdivided in two columns according to each flanker mean: 6 degrees (white background), 18 degrees (gray background). Panel A. No-mask condition (blue). Panel B. Mask condition (red).

There were two main effects found in the saccade condition. First, as found in Experiment 2, there was a general benefit of making a saccade in the no-mask condition (Fig 5A). This effect was significant when the mean flanker orientation was equal to 6° (95% CI, [0.05 0.56]) and 18° (95% CI, [0.15 0.64]).

More interestingly, we found that during no-mask trials, discrimination performance actually decreased significantly along the pre-saccadic interval in the 6° mean orientation condition (95% CI: [-0.34–0.04]). On these trials, participant judgments appeared to follow the orientation of the entire configuration, rather than the target, during the peri-saccadic interval. In the masked condition, there was no significant change in the slope (Fig 5B), where performance was always around chance level. From the present data it is difficult to have a strong interpretation for the lack of effect with masks, but there are two important caveats to take into consideration. First, we can observe that even in the fixation condition at 2dva of TFD (Fig 4C and 4D) the same participants showed very little (no-mask) or no modulation (mask) along the flanker mean dimension. This observation alone raises the question of whether there was a floor effect in Experiment 3B for those conditions and required further testing.

Second, it is important to note that spatial compression effects are strongest for briefly flashed items, with spatial compression dramatically reduced, or even eliminated, for stimuli with longer durations of stimuli or for stimuli presented in a sequence, as in the masking condition here [53], [54]. This might explain why increased pooling (influence of the average orientation of the flankers) was found in the no-mask condition, whereas in the mask condition there was little effect of the saccade either in the positive sense (performance always near chance, so no un-masking) or the negative sense (no drop in performance along the time course of the saccade). Consistent with this idea, it has recently been shown that adding forward and backward masks reduces mis-localization but does not eliminate a more general pre-saccadic benefit in performance [54].

The results for the no-mask condition are consistent with averaging of the flanker orientation with the target (pooling) during the pre-saccadic interval. Such pooling would be consistent with the neurophysiological [24], computational [55] and behavioral [56], [57], [58], [59], [60] reports that space is effectively compressed towards the saccadic target location just before saccades. Interestingly, the time course of the pooling seemed to be independent from the earlier, and more general benefit given by the eye movement preparation, as found also in Experiment 1B. In order to better characterize this potential dissociation in the time courses of a general covert attention shift effect and a pre-saccadic pooling effect, we ran a final experiment (Experiment 4). To be more sensitive to spatial compression effects [53], [54], we only used the no-mask condition in the final experiment.

## Experiment 4

A critical question in theories of visual stability is the role of the saccadic target. At a theoretical level, it has been suggested that visual updating is mainly focused on the saccade target [61], [62]. The close link between selective attention and saccades, as well as the finding of spatial compression in behavioral and neurophysiological data, would suggest that both the enhancement and the pooling effects found here might be limited to the saccade target. In contrast, other studies have demonstrated trans-saccadic updating for non-target objects (for review, see: [63]), with the proposal that multiple items in visual working memory (VWM) are updated across saccades (for review, see [64]). Spatial remapping of receptive fields has also been demonstrated for other, non-saccade-target locations, which might benefit (in the case of remapping against the direction of the saccade) or hinder (in the case of compression towards the saccade target or in the direction of the saccade) performance.

**Fig 6:**
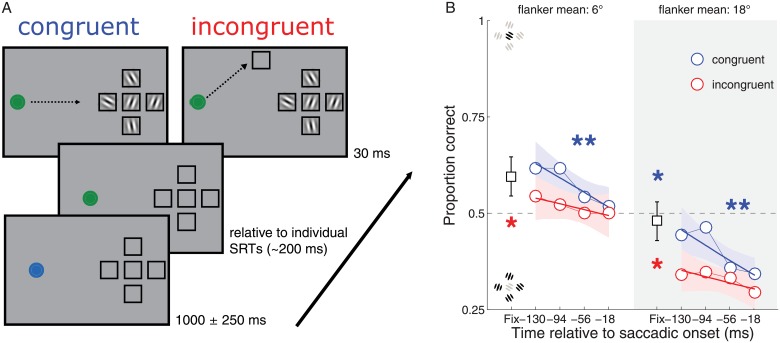
Design and results for Experiment 4. Panel A. In Experiment 4, during incongruent blocks, an empty placeholder was located 40° away from the horizontal plane, at the same eccentricity as the perceptual target, to serve as saccadic target. In the current figure, a no-mask trial is depicted with the flanker orientation randomized and its mean orientation pointing to the left. Target orientation is instead pointing to the right. Panel B. Data for Experiment 4 are organized according to flanker mean: 6 degrees (white background) and 18 degrees (gray background). The congruent condition is displayed in blue while the incongruent condition is depicted in red. The black square represents mean the performance at fixation and its 95% CI. Data are displayed relative to saccadic onset. All other graphical conventions (lines, asterisks) are identical to those used in Fig 2.

We investigated the spatial selectivity of the peri-saccadic effects found in the first three experiments by including trials in which the discrimination target was either congruent or incongruent with the saccadic target (Fig 6A). Based on previous studies of pre-saccadic enhancement and attention, we would predict that the pre-saccadic benefit should be limited to the saccade target [6], [7], [8], with the enhancement at the target complemented by a decrement for a non-saccade-target location compared to no-saccade trials. However, we hypothesized that a bias towards the mean flanker orientation might occur for both congruent and incongruent locations due to compulsory pooling and/or shifting receptive fields towards the saccade target.

## Experiment 4: Methods

### Procedure

We compared a condition when the perceptual target and the saccadic target were at the same spatial location (“congruent”) with a condition in which the saccadic target rotated by 40° away from the perceptual target (“incongruent”) (Fig 6A). All the other stimuli and timing were identical to the Experiment 3 no-mask condition. In the *congruent* condition, the perceptual target was at the same location as the saccadic target, as in Experiment 3. In the *incongruent* condition, however, an empty placeholder was located 40° of angle away from the horizontal plane, at the same eccentricity as the perceptual target. This empty placeholder served as saccadic target on blocks in which a saccade was required, dissociating the saccadic target from the discrimination target in a similar way to the classical procedure used by Deubel and colleagues [6], [9]. On some blocks, participants maintained fixation and reported the orientation of the Gabor presented at the center of the configuration. For the fixation session, each participant performed two blocks of 80 trials. In the other blocks, participants made an eye movement either towards the discrimination/perceptual target or to the empty placeholder while also reporting the orientation of the Gabor patch (discrimination target) presented at the center of the configuration. Participants performed 9 blocks of trials. Each block consisted of 80 trials (40 congruent and 40 incongruent trials), for a total of 720 trials for each participant. As in Experiment 3, within each block, there were 12 catch trials. Fixation and saccade blocks were counterbalanced across participants according to an ABBA sequence. Fixation trials in which participants made an eye movement larger than 1 dva were excluded (<2%) and saccade trials in which there were saccades with a latency of less than 80 ms or more than 500 ms and with amplitudes smaller than 4.5 dva were excluded (~22%).

## Experiment 4: Results

As it is possible to appreciate in Fig 6B, we replicated the finding from Experiment 3B that discrimination performance was reduced during the peri-saccadic time period along the time course leading up to saccade onset. This effect was clear for both experiments when the mean flanker orientation was equal to 6°, while it was reliable in Experiment 4 also for a flanker mean of 18°. In particular, near to the time of the saccade, participants tended to report the mean orientation of the flankers, not the target orientation. Although the slope was consistently negative across all four conditions, this pooling effect reached significance only for the two congruent conditions (95% CI, [-0.28–0.02] and [-0.28–0.04], for the average flanker orientation of 6° and 18°). The results were similar for both the conditions with 6° and 18° average flanker mean, supporting and expanding the finding of Experiment 3B.

There was also a more general influence of congruency on performance, with consistently worse performance in the incongruent condition compared to both the fixation and congruent conditions. This was evident by comparing the performance during the pre-saccadic interval between the congruent vs. incongruent condition (95% CI, [-0.59–0.38]). Thus, in addition to showing the benefit on performance, well before saccade onset, for the saccade target in the previous experiments, we now show also the concurrent reduction in performance at a non-target location starting in the earliest pre-saccadic time bin. Together, this provides converging evidence that enhancement at the saccade target, with respect to fixation and with respect to a non-target item, occurs early and is specific to the saccade target location. Moreover, the results of Experiment 3B and this experiment suggest that the time course and effect of the enhancement tied to saccades shows a different time course to the pooling effect, which is instead strongest later in time, near saccade onset. This follows the consistent finding that spatial compression also peaks around saccade onset (for review, see: [32]).

## General discussion

According to active perception and sensorimotor theories, visual processing and eye movements are closely linked and work together to yield continuous, stable perception that can guide our actions (for review: [22], [65], [66]). In terms of psychophysics, evidence for this tight link comes from studies of changes in performance around the time of saccades as well as a number of studies showing non-retinotopic visual perception (for review: [22]). Likewise changes in the spatial and temporal tuning of receptive fields, as described above, as well as phase resets in neural oscillations [66], [67], [68], point to a process by which visual perception and saccades are actively aligned [66].

In the current study, we investigated the time course of the mechanisms that lead to pre-saccadic enhancement, which have been shown to include a change in feature tuning at the saccade target [12], as well as peri-saccadic changes in spatial tuning, including spatial compression. Our results are consistent with recent neurophysiological evidence [19], [20] that these two mechanisms have a different time course, with enhancement of the saccade target happening earlier (Experiment 1B) and maximum changes in spatial tuning (spatial compression) happening closer to saccade onset (Experiments 3B and 4).

The current findings, together with previous studies, suggest that there are two main factors that make peripheral stimuli in a complex environment seem subjectively available to our perception. First, as soon as we are interested in looking at an item, our focus of visual processing shifts very quickly, much faster than our gaze, to the target of interest and influences our ability to discriminate its features. It has been suggested that, in a certain sense, the entire near periphery, which can be reached with a single saccade, may seem subjectively to be clearly perceived because it is almost immediately available to us in high detail via saccades [69]. Indeed, the illusion may be even more compelling due to the fact that this enhancement of the saccade target occurs even well before the saccade onset. The current findings help to better characterize the time course of this effect, showing that it is strong and consistent prior to the saccade itself.

Second, our results provide further evidence for a spatio-temporal transformation that begins before the saccade and peaks immediately before saccade onset and might be related to the phenomenon of spatial compression around the saccadic target (for review: [65], [70], [71]). In contrast to the first mechanism, which effectively involves a specific benefit at the saccade target, the second mechanism involves spatial and temporal changes that influence perception in a more complex way and with a different temporal profile.

As shown in Experiments 3 and 4, while the first mechanism (enhancement) would tend to improve discrimination of the target versus distractors, the second mechanism (compression) might instead enhance a more configural, ensemble-like, or statistical representation of the visual features near the saccade target [72], [73]. Given that saccades are neither highly precise nor accurate in everyday viewing, using information near the saccade target to predict what will be seen after the saccade might actually be more effective than only taking into account the saccade target itself [65], [74]. In our study, for example, subjects did consistently land somewhere on the configuration of target and flankers but not always exactly on the target. Saccadic landing position spread and undershoots are consistently reported across eye movement studies. Therefore, it might be the case that the optimal strategy for predicting [75], [76] and integrating [77], [78] (for review: see [22]) visual features such as orientation across saccades would be to take into account the distribution of features in the entire potential landing area.

It is important to note that we did closely replicate a previous report of better performance in a task using crowding (and in particular crowding followed by a mask) prior to saccades [27], although our explanation of these results differs and follows instead the conclusions of Ağaoğlu and colleagues [37] (see also: [36]). In particular, both the fixation condition (using masks) at different target-flanker conditions (Experiment 1A and 2A, Figs 2 and 3) and the pre-saccadic improvement in performance for target-flanker separation of 2 degrees are nearly identical to a previous study [27]. Our analyses of the time course of these effects, however, indicated that the strongest and most consistent effect was actually between the no-saccade condition and the saccade condition. When not including the fixation condition in the fit of the trend line, discrimination performance did not increase significantly along the peri-saccadic interval. Thus, an important difference between our study and earlier reports in interpreting the underlying mechanism comes from a difference in how the data were fitted in order to appreciate the full time course of the effect. Other than different interpretation of the data because of different type of statistics used, we would point out that our results more generally do not support saccadic un-crowding, and consequently we suggest that pre-saccadic performance improvements for crowded stimuli are mostly due to enhancement and saccadic unmasking.

Moreover, due to our novel introduction of the manipulation of flanker orientation, we found that spatial pooling actually increased along the peri-saccadic time period for non-masked stimuli. At present, there are a large number of physiological [13], [14], [15], [16], [17], [18], [24] and behavioral [32], [33], [57] studies showing spatial compression towards the saccade target, and this could account for the increase in pooling found here. The finding that this tendency to base judgments on the mean flanker duration tended to increase closer to saccade onset, as well as the fact that it was found only for non-masked stimuli, is consistent with the finding that spatial compression is also strongest right before the saccade and only occurs for brief stimuli. This similarity is suggestive of a link between these two phenomena, but further studies would be needed to confirm this possibility.

A recent paper by Ağaoğlu and colleagues [37] also concluded that the main influence of saccades in a similar paradigm was un-masking, rather than un-crowding (see also: [36], [79]). In one key condition, they reported similar effects of the saccade on performance with and without flankers. However, that study used a different stimulus (letters), raising the question of whether the difference between their findings and those of Harrison and colleagues [27] was due to different levels of stimulus processing. Ağaoğlu and colleagues [37] also found a small enhancement on saccade trials, similar to that found here. Moreover, in a recent study, Ağaoğlu and Chung [38] tested the influence of eye movements on crowding with different combinations of high/low contrast target/flanker stimuli and did not observe any evidence of a release of crowding in any of the conditions tested. In their paper, the authors supported a framework whereby the pre-saccadic modulations are mostly driven by spatial factors, such as localization of the stimuli and saccadic suppression. Our design was not sensitive to saccadic suppression effects, since we used high contrast stimuli and the benefit in performance was found quite early and did not increase during the time period that suppression might have reduced the effectiveness of the post-target mask [34]. The reduction in performance near saccade onset was only found for flankers with random orientation, and this specificity is difficult to reconcile with suppression. In the current study, by manipulating the flanker orientation we were able to show a significant decrement in performance during the pre-saccadic time course, which would be consistent with the modeling results from Ağaoğlu and colleagues that incorporated multiple mechanisms, rather than a pre-saccadic release from crowding [27].

In conclusion, this investigation of the perception of crowded and masked stimuli around the time of saccades suggests that it is a useful strategy to try to disentangle the mechanisms and time courses of pre-saccadic enhancement from peri-saccadic changes in spatio-temporal processing due to spatial compression. Critically, both mechanisms rely on the ability to predict the upcoming oculomotor plan, consistent with a role for the efference copy (for review, see: [71]). The interplay of enhancement and spatial compression towards the saccade target may underlie our subjective experience of continuous and stable perception of the world, despite the fact that the position of objects on the retina changes several times per second due to our own body movements and object movement.
